# Examining Similarity Structure: Multidimensional Scaling and Related Approaches in Neuroimaging

**DOI:** 10.1155/2013/796183

**Published:** 2013-04-15

**Authors:** Svetlana V. Shinkareva, Jing Wang, Douglas H. Wedell

**Affiliations:** Department of Psychology, University of South Carolina, Columbia, SC 29208, USA

## Abstract

This paper covers similarity analyses, a subset of multivariate pattern analysis techniques that are based on similarity spaces defined by multivariate patterns. These techniques offer several advantages and complement other methods for brain data analyses, as they allow for comparison of representational structure across individuals, brain regions, and data acquisition methods. Particular attention is paid to multidimensional scaling and related approaches that yield spatial representations or provide methods for characterizing individual differences. We highlight unique contributions of these methods by reviewing recent applications to functional magnetic resonance imaging data and emphasize areas of caution in applying and interpreting similarity analysis methods.

## 1. Introduction

Researchers who engage in neuroimaging methods face many daunting challenges associated with the vastness and complexity of the data gathered in even a modest experiment with few participants. These data can be analyzed at several different levels, each of which may serve a different theoretical purpose. Recent methodological advances in multivariate pattern analyses (MVPA) have shifted the focus from examining the responses of individual voxels to examining patterns of neural activity associated with different cognitive processes and mental representations (for reviews of MVPA approaches, see [1–4]). This paper covers a subset of MVPA techniques that are based on similarity spaces defined by multivariate patterns. These methods, which include multidimensional scaling and representational similarity analyses, have been gaining increasing popularity in the neuroimaging literature. Applications of these methods range from the examination of the internal representation of objects (e.g., [5]) to the examination of the functional connectivity of different brain regions (e.g., [6]). 

Similarity based methods are very flexible. A variety of methods can be used to construct a pairwise similarity matrix to represent the proximity relationships among the entities of interest. In fMRI research these entities are often voxel activation patterns associated with the corresponding states or cognitive representations elicited by presentation of different stimuli, tasks, or conditions. Additionally they may correspond to different brain regions or even individuals themselves. While many MVPA methods use patterns of activity to classify different states or representations, similarity based methods examine the relationships among those patterns to make inferences about relationships in the data at the neural, cognitive, or behavioral levels of analysis. These methods provide valuable insights into processes and representations that may be inferred from the data. They have received a considerable interest in recent neuroimaging literature, as seen from numerous applications, as well as methodological advances [7–10]. For example, Kriegeskorte and colleagues [7] have proposed representational similarity analysis (RSA) as a framework for comparing activity-pattern dissimilarity matrices generated by different data gathering methods, such as behavioral and neuroimaging. Some of the advantages of these analytic methods have been discussed by Connolly et al. [11]. The goals of this paper are to introduce similarity analyses to the broader neuroimaging community, highlight the advantages of abstracting from activation patterns to the similarity structure among these patterns, and illustrate the utility of these techniques by reviewing recent applications. We will focus on fMRI data, although the methods are equally applicable to other neuroimaging modalities as well, such as MEG or EEG.

Similarity analyses have a long history of wide-ranging applications in the sciences. For example, multidimensional scaling (MDS) has been used to visualize data in such diverse fields as psychology, biology, geography, marketing, sociology, physics, and political science. Many applications in psychology have been directed toward understanding perceptual and conceptual representations and processes associated with interobject similarity (e.g., [12–15]). An advantage of similarity analyses is that they can take place at many different levels. For example, neural representations may be compared through the analysis of differences between neural activation patterns [16]. More broadly, decoding across individuals may be considered to take place within a shared similarity space, with commonalities and differences in the similarity matrix of each individual used as input for further analysis [9]. While the focus of visualization techniques, such as MDS, is primarily to derive a spatial representation of entities being compared (e.g., stimuli, states, and neural regions), other techniques, such as RSA, can provide a comparison across brain-activity measurements, behavioral measurements, physical measurements, and computational modeling at a level of dissimilarity matrices [7]. Thus, these methods are both flexible and general. We begin with outlining the advantages of similarity analyses. We then discuss the data used with these techniques and describe two multivariate methods for visualization of similarity structure: multidimensional scaling and cluster analysis. We conclude by reviewing current applications of similarity analyses in neuroimaging. 

## 2. Similarity Analyses

### 2.1. Advantages

When used in conjunction with MVPA methods, the examination of similarity relationships offers several advantages over simply focusing on activation patterns of conditions directly. Analyzing the similarity structure of activation patterns allows one to evaluate hypotheses without specifying brain regions or locations [7, 17]. Moreover, the individuals' data are compared at the level of similarity matrices generated from response patterns, thus allowing for different spatial correspondences of individual patterns as well as different number of variables (e.g., voxels) per individual. An additional advantage is that these methods do not require spatial normalization of the individual's data [1, 9, 10, 18]. The similarity matrices can be directly compared across people, brain regions and data collection methods. 

The flexibility of similarity based methods allows for comparison of internal representations derived from fMRI data to those based on behavioral responses, computation, or physical characteristics of stimuli. These comparisons can ground hypotheses about neural representations [1] and form the basis of RSA [7]. Furthermore, these methods allow for comparison of similarity matrices across individuals to examine the consistency of internal representations [1, 10]. 

The construction of similarity measures of activation patterns between conditions, instead of distributed patterns themselves, has been successfully used for object decoding across individuals [9]. In cases when assumptions of additivity and linearity are not met, similarity based methods still provide for comparisons based strictly on ordinal relationships. Additionally, examination of similarity structure allows for abstract depiction of representations using multivariate techniques such as MDS and cluster analysis. Thus, similarity analyses are flexible and can be incorporated into many different types of analyses as well as used for comparisons across people, brain regions, and data collection methods.

### 2.2. Data

We will refer to the entities under investigation as objects, for consistency with the multidimensional scaling literature. For example, objects can refer to stimuli, brain regions, or individuals. Similarity analyses focus on the object-by-object matrix of proximities, a generic term that refers to either similarity or dissimilarity. The *ij*th cell of the proximity matrix is a proximity value for the pair of objects *i* and *j*. For behavioral data, proximities can be either collected directly or derived. However, for neuroimaging data, proximities among pairs of objects are typically derived from comparing patterns of pairwise brain activity. When activity patterns match, distance is minimal, and similarity is maximal. The degree of match is often measured by simple correlation. An alternative method for computing proximity between objects is to use confusability derived from classification models. We begin by discussing the formation of multivoxel patterns. 

#### 2.2.1. Multivoxel Patterns

 Each object can be represented by a multivoxel pattern of brain activity values. These patterns of activity can be viewed as points in a multidimensional space with dimensionality equaling the number of voxels. Multivoxel patterns of activity can be either estimated or extracted from neuroimaging data. The reader may benefit from general discussions of data used for MVPA [19, 20]. Functional activity corresponding to a single object for each voxel can be estimated using general linear modeling (e.g., [21]). In that case the pattern of activity for each object will consist of beta values. The advantages of this approach are the ability to include nuisance regressors into the model and to deal with overlapping hemodynamic responses. Alternatively, in cases when the trials are minimally overlapping, the pattern of activity can be formed by single or temporally averaged normalized signal intensity values (e.g., [10, 22–28]). 

#### 2.2.2. Feature Selection

 Once the multivoxel activity pattern has been abstracted from the data, one has to decide which voxels to include in the analysis. The total number of voxels is typically large, and inclusion of voxels that are not relevant introduces noise that will obscure the systematic relationships in the data. There are several possibilities, and a particular choice largely depends on the application area. Analyses often focus on theoretically motivated regions of interest (ROI) and may additionally be constrained by further criteria. For example, Kriegeskorte and colleagues [21] examined object representations in inferior temporal cortex with an additional feature selection based on visual object responses from an independent data set. Other approaches have been to restrict the analyses to gray matter voxels [10] or to those voxels whose activation exceeds a certain threshold not related to the contrast of interest [5]. Another possibility is to limit the data to a smaller number of principal components, relative to the number of voxels. It is critical that voxel selection criteria do not bias the results; otherwise the results may simply be an artifact of the selection process. Once selection has taken place, each object is represented by the multivoxel pattern of activity, and pairwise proximity values can be computed.

#### 2.2.3. Proximities

 There are several ways to measure proximities between pairs of objects. Generally, measures of dissimilarity (e.g., distances) are used to compare items, and measures of similarity (e.g., correlation) are used to compare variables. Proximities for each pair of objects are organized into a square matrix of proximities. A proximity matrix is assumed to be symmetric with minimum distances (or maximum similarities) on the diagonal. When proximities are calculated from patterns of activities, these assumptions generally hold. 

In summary, proximities are easily computed with neuroimaging data based on correspondence between pairwise activation patterns. One difficulty lies in the selection of relevant input variables (i.e., voxels), as the inclusion of large numbers of irrelevant variables will typically mean that relevant proximities are obscured by noise. Extreme caution should be exercised in choosing unbiased variable selection criteria.

In the neuroimaging literature, there are examples of proximity matrices created from activation patterns or confusability patterns. Proximity for a pair of objects has been defined as pairwise Euclidean distances [5, 6, 29], correlation distances, computed as one minus the Pearson correlation [7, 30], one minus the absolute value of the partial correlation [31], as well as Pearson correlation [9, 32–34] computed between activation patterns for pairs of objects. Other researchers have used the absolute difference of responses between conditions [35] or the squared deviation of responses [36]. Proximity matrices can also be constructed from confusability patterns generated from classification models. For example, several studies that examined classification between objects have used the information based on the frequency of correct and misclassification results [1, 29, 36–38]. In a similar vein, Greenstein et al. [39] defined the proximity between participants by how often they were classified to the same group. Shinkareva et al. [22] compared brain regions in terms of the confusion patterns based on the most likely prediction of the classifier for object classification.

### 2.3. Direct Comparison of Similarity Structures

Objects × objects proximity matrices can be derived from many different sources. For example, they may correspond to different brain regions, individuals, or data collection methods. The relatedness of two proximity matrices can be evaluated with a correlation coefficient and tested by randomization [7]. The rows and columns of one of the matrices can be permuted, and the correlation can be recomputed. The procedure can be repeated a large number of times, simulating the distribution of the correlation coefficient under the null hypothesis of no relationship between the two matrices. The observed correlation coefficient can then be compared to the permutation distribution of correlation coefficients. For readings on additional tests for comparison of correlation matrices and their elements the reader is referred to Steiger [40].

## 3. Visualization of Similarity Structure

We discuss two sets of exploratory multivariate techniques that are commonly used in neuroimaging applications for visualization of similarity structure, multidimensional scaling, and cluster analysis.

### 3.1. MDS

Multidimensional scaling is a set of techniques for analysis of proximities (similarities or dissimilarities) that reveals structure and facilitates visualization of high dimensional data. MDS has a long history in psychology and neuroscience and has been used extensively for analyzing behaviorally derived data (e.g., [41]) and single-cell recordings data (e.g., [42–47]). Multidimensional scaling seeks to find a lower dimensional representation for a set of *n* objects (e.g., stimuli, brain regions, individuals, etc.) by representing the interobject proximities as distances in some lower dimensional space. We give a brief account of MDS. There are a number of good references that provide a more in-depth overview of MDS for the interested reader [48–50]. 

Assume that a measure of proximity (*p*_*ij*_) is given for every pair (*i*, *j*) of *n* objects. The proximity matrix is assumed to be symmetric with nonnegative dissimilarity values. Thus for *n* objects there are *n*(*n* − 1)/2 proximities. Let *X* be an *m*-dimensional configuration of objects, such that *m* < *n*. The mapping from proximities to distances is accomplished through a representation function, *f*, which specifies how the proximities should be related to distances, *f* : *p*_*ij*_ → *d*_*ij*_(*X*), where the distances (in an Euclidean model) are computed as *d*_*ij*_(*X*) = [∑_*a*=1_^*m*^(*x*_*ia*_ − *x*_*ja*_)^2^]^1/2^. It is a special case of a general distance measure, a Minkowski metric, defined as *d*_*ij*_(*X*) = [∑_*a*=1_^*m*^(*x*_*ia*_ − *x*_*ja*_)^*k*^]^1/*k*^. For *k* = 1, *d*_*ij*_(*X*) measures the *city-block* distance in *m* dimensions, and for *k* = 2, *d*_*ij*_(*X*) measures Euclidean distance in *m* dimensions. In general, choice of *k* changes the weight for larger and smaller differences. When *k* is 1, the spatial solution cannot be rotated without changing underlying distances. It has been shown that perceived similarities for stimuli that are not perceptually analyzed into separate features conform with a Euclidean metric, while perceived similarities for stimuli that vary along perceptually distinct dimensions depend on subject's state of attention and are better described by a city-block metric [51]. For MDS the Euclidean metric is often chosen as the solutions that are robust and not as limited by problems of local minima [52, 53].

MDS attempts to find a configuration *X* that satisfies *f* as closely as possible. The choice of *f* specifies the MDS model. In metric MDS the dissimilarity data are assumed to be measured on a ratio or interval scale. In the behavioral literature, metric MDS is often used to determine starting values for minimizing distances; however, it is rarely used as a final model because the assumption of metric data is typically not satisfied. Instead, the most common assumption is that proximities are measured on an ordinal scale. The rank order of proximities between objects can be used to determine the dimensionality of the space and metric configuration of the points representing the objects [54, 55], referred to as nonmetric MDS. While proximities are assumed to only be ordinal, the resulting distances are assumed to be measured at a ratio level.

Determining the MDS solution is typically an iterative process in which the badness-of-fit measure for the MDS representation, called stress, is minimized. The objective function that is minimized is a normed sum-of-squares of representation errors, *e*_*ij*_ = *f*(*p*_*ij*_) − *d*_*ij*_(*X*). A useful tool for visualizing the fit of the model is the Shepard diagram, which plots proximities *p*_*ij*_ against the corresponding distances *d*_*ij*_ and modeled distances ${\hat{d}}_{ij}$. When the scatterplot in a Shepard diagram is well approximated by a linear function, then metric MDS is appropriate. When Euclidean distance is assumed, the MDS solution is indeterminate with respect to translation, rotation, and reflection. For interpretation purposes, properties of the objects measured on unidimensional scales may be regressed onto the solution and plotted as vectors in the MDS space [48]. To compare solutions, a Procrustes rotation may be applied to match the orientation of the configuration as closely as possible to a fixed design matrix without distorting distance information [56]. 

#### 3.1.1. Assessing Fit and Selecting the Number of Dimensions

There is no statistical test for selecting the correct number of dimensions. Typically researchers conduct the MDS analysis for several successive values of the number of dimensions and select the solution that seems most appropriate. A plot of dimensionality versus fit, called a scree plot, is useful in selecting the appropriate number of dimensions when there is a clear elbow. Increasing the number of dimensions reduces stress values. Choosing too many dimensions results in over fitting the data, so that the configuration reflects unstable influences of noise. For *n* objects, zero stress value can be obtained for *m* ≥ *n* − 1 dimensions; however, this solution is undesirable. On the other hand, choosing too few dimensions may result in the true structure being distorted. Ultimately, the interpretability of dimensions is an important factor in deciding on the number of dimensions, as uninterpretable dimensions are not useful.

#### 3.1.2. Number of Stimuli

There are a number of factors to consider in deciding on the number of stimuli to use in a prospective study or the appropriateness of using MDS on a given data set. The number of fitted dimensions depends on the number of stimuli, as a perfect solution may be achieved with *n* − 1 dimensions. For instance, four points may be perfectly represented in three dimensions. When distances are inferred from ordinal relationships, a relatively large number of stimuli are required, so that accurate distance information may be derived. Finally, it is useful to have multiple stimuli of the same type to demonstrate that these fall in similar locations within the MDS space, helping to gauge the reliability of the inferred representation.

#### 3.1.3. MDS with Multiple Matrices

The previous basic algorithm was presented for a single matrix of proximities (two-way data: objects × objects). However, most neuroimaging data is collected for a group of individuals, and so a methodological question arises concerning how to aggregate individual proximity matrices into a single analysis. If little commonality exists between individual proximity matrices, aggregating the data is not meaningful, as the average will not represent any of the constituents. On the other extreme, if the differences between individual proximity matrices are not systematic, interpreting the differences is not meaningful. Most data sets, however, lie between these two extremes. Each proximity matrix can be analyzed separately, although it is difficult to summarize the results for a group of individuals or compare the results across groups. Moreover, additional data may be needed to obtain stable results for an individual [57]. Another approach is to analyze the mean proximity matrix and to generalize the results to an “average” individual (e.g., [31]). This approach is straightforward, but the results may not accurately capture the consistent relationships shared by individuals, and all individual differences will be lost [57]. 

Several algorithms have been proposed to simultaneously analyze multiple proximity matrices (three-way data: objects × objects × individuals). These approaches offer two key advantages over analyzing each proximity matrix individually or averaging the matrices together. First, in cases when individual proximity matrices are noisy, these methods take advantage of commonalities among individuals. Second, group space provides a useful basis for comparison of individuals [58]. We review two sets of techniques that have been extensively used in neuroimaging literature: individual differences scaling [25, 33–35, 59, 60], an iterative set of techniques, and STATIS [1, 10, 22, 61–63], an eigen decomposition based set of techniques. 

The most popular algorithm for individual differences scaling is INDSCAL [58]. It assumes that some number of dimensions, *m*, is common to all individuals. Individuals are assumed to differentially weight the several dimensions of a common space, such that the effective distance between objects *i* and *j* for individual *s* is *d*_*ijs*_(*X*) = [∑_*a*=1_^*m*^ *w*_*sa*_(*x*_*ia*_ − *x*_*ja*_)^2^]^1/2^, where *w*_*sa*_ is the weight of dimension *a* for individual *s*. Thus the weights represent the relative importance that each individual places on a given dimension. Nonlinear iterative least squares are used to obtain a metric configuration. Unlike the MDS solution based on a single proximity matrix discussed previously, the INDSCAL solution is not rotation invariant. The output of the algorithm is an objects-by-dimensions matrix of coordinates defining the group space and participants-by-dimensions matrix of weights defining individual spaces. The group space represents an individual that places equal importance on all the dimensions. In cases where groups of individuals all have different patterns of weights, the group space is merely a compromise configuration and may not be representative of any of the individual matrices. The model accounts for individual differences in terms of differential salience of a common set of dimensions. Each individual's space is estimated by weighting a common set of dimensions in the group space. Because INDSCAL is a dimensional model, it is inappropriate for clustered data, where values do not differ continuously along a dimension. One limitation of the approach is that individual spaces in INDSCAL are related by linear transformation of a common space along the specified dimensions, and nonlinear distortions of a common space may require too many dimensions [58]. 

STATIS, which stands for *Structuration des Tableaux à Trois Indices de la Statistique,* is a generalization of principal component analysis for multiple data matrices [64]. Individuals' data are combined into an optimum weighted matrix called a compromise. The weights of individuals' data are chosen, such that the compromise is as representative of all the data as possible. Thus, the compromise matrix expresses the agreement among the interobject distances across individuals and is constructed, such that individuals with configurations of objects similar to those of other individuals are assigned larger weights, and individuals with configurations of objects most different from those of others are assigned lower weights. As a consequence, unusual or atypical observations have less influence on the result. The compromise matrix is further analyzed by the eigen decomposition to reveal structure that is common across individuals. Individual data can be projected into the common compromise space. STATIS works with individual objects-by-variables data matrices. COVSTATIS and DISTATIS are generalizations of STATIS for objects-by-objects covariance and distance matrices, respectively. Thus, like individual differences scaling, STATIS indexes the relative contribution of individuals to a common configuration, but it does so by differentially weighting individual matrices instead of fitting differential dimension weights across individuals. For in-depth treatment of the STATIS procedure the reader is referred to Abdi and Valentin [65] and Abdi and Williams [8]. 

### 3.2. Cluster analysis

Cluster analysis seeks to discover natural nonoverlapping groupings of objects. Hierarchical clustering techniques are perhaps most popular in neuroimaging applications for similarity structure visualization. Hierarchical clustering techniques produce a nested sequence of partitions and can be either agglomerative or divisive. In agglomerative hierarchical clustering each object starts out in its own group. In a series of successive mergers similar objects get grouped together until finally all objects are grouped together. Divisive hierarchical methods operate in an opposite direction. Types of hierarchical clustering vary on how the similarity is defined for groups of objects. For instance, average linkage computes an average distance, complete linkage computes maximum distance, and single linkage computes minimum distance between clusters. Once objects have been grouped together in hierarchical clustering, they cannot be regrouped. Hierarchical clustering results are visualized with a dendrogram, a tree diagram showing successive groupings of the objects. Selecting a partition is thus equivalent to cutting the dendrogram at a given height. The clustering results depend both on choice of proximity and linkage methods. A challenging decision in cluster analysis is to select a number of clusters and to check the validity of the solution. For a detailed treatment of the cluster analysis the reader is referred to Johnson and Wichern [66], Arabie et al. [67], and Landau and Ster [68]. 

## 4. Applications

 We will next review the applications of similarity analyses in fMRI literature. Both MDS and cluster analysis have been used as exploratory tools for visualization of similarity structure derived from fMRI data. Additionally, RSA has been used to test hypothesized relationships between similarity matrices. We will group the studies based on entities under investigation: stimuli (focusing on internal representations), individuals, and brain regions. 

### 4.1. Similarity of Internal Representations

Object representation in the brain has been extensively studied with the aid of similarity analyses of single-cell recordings data (e.g., [42–47]) and, to a lesser extent, fMRI data [69] in monkeys. Here, we review the role of similarity analyses in examining the internal representation of objects from fMRI data in humans. Edelman and colleagues [5] used MDS to visualize the internal representation of objects based on distributed patterns of voxel activity in the human visual cortex. They found a close association between the representational space of object categories derived from fMRI data and that derived from perceptual similarity judgments. Furthermore, the internal representation of shape using novel objects derived from fMRI data in the lateral occipital complex (LOC) was shown to be similar to the subject perceptual similarity space but less so to the pixelwise physical space [33]. The representational similarity in posterior LOC was found to correlate with the physical stimulus similarity, while the anterior LOC correlated with the perceived similarity [70]. Such physical-to-perceptual shifts along the ventral visual pathway were also found in the visual perception of different texture types [29]. Thus, by examining similarity structure derived from neural data in different regions, the resulting configurations could be compared to similarity structure derived from physical stimuli (pixelwise space) and similarity structure derived from perceptual judgments (perceptual space) to show how these representations were captured by different areas in the brain.

Object representation across different tasks has been examined by Tzagarakis et al. [35] who used individual differences scaling with multiple cortical areas to investigate neural mechanisms associated with viewing and copying geometric shapes in the absence of visual feedback. Using predefined features, the study identified different perceptual and motor features of geometric shapes that were associated with the dimensions in MDS solutions for viewing and copying tasks. The extent to which each cortical area contributed to the dimensions could also be identified. In this investigation of object representation, INDSCAL was used to compare representations across different brain regions.

Representation of objects that come from different categories has also been examined. O'Toole et al. [1] used the DISTATIS method on fMRI data in the ventrotemporal cortex collected while viewing pictures from different object categories [71] and have showed that the internal representation of objects derived from fMRI data is consistent across participants and is similar to physical space. Shinkareva et al. [10] used STATIS to examine the commonality of object representation across individuals and have showed consistency of separation for tools and dwellings categories across individuals. Thus, in the object domain, dimensional visualization techniques have proven useful in comparing representations across individuals. 

In an investigation of category structure of objects, Op de Beeck et al. [34] used MDS to visualize the perceived shape similarity and the neural representational similarity spaces for six subordinate categories of objects from human faces, human bodies, and buildings. Results showed that although the hand images were distinct from other categories in the perceived shape space, two clusters of human body/head and buildings were identified in the neural space. Similarity of neural activity patterns in various regions in the ventral visual pathway was found to be correlated with behavioral similarity ratings on pictures of 18 mammals [36]. Connolly et al. [30] investigated subordinate relations of objects within the animate domain in the ventrotemporal cortex and have shown that patterns of activity reflect the biological classes of the stimuli. Moreover, the cortical representational similarity was also correlated with behavioral judgments of biological similarity of the same stimuli. Converging evidence came from cluster analysis on the trained classifier for multivoxel pattern analysis [72]. Cluster analysis on the trained hidden units of a neural network classifier of eight object categories revealed distinctions among the eight categories as well as the distinction between two animate and six inanimate categories. These results suggest an animate-to-inanimate gradation represented in the ventral temporal cortex. 

Some of the aforementioned studies have used representational similarity analysis and compared the internal representation derived from fMRI data to another representation. The internal representation of objects derived from fMRI data has been compared to internal representation derived from perceptual space [1, 5, 29, 33–35, 70], stimulus parameters or image-based physical similarity measures [29, 33, 70], or subjective conceptual similarity ratings [36]. Perhaps the most elegant application of representational similarity analysis is an investigation of object representation in inferior temporal cortex in monkeys and humans showing that the internal representation of objects is similar across species [21]. The authors used both multidimensional scaling and hierarchical cluster analysis to explore whether the inferior temporal cortex response patterns form clusters corresponding to natural categories for human and monkey, in data sets collected in independent experiments. They have shown separation for animate and inanimate classes, as well as faces for both humans and monkeys. 

In addition to object representation studies, MDS has been used to examine the internal representation of affective states [25, 73], internal organization of the visual word form area [74], and categorization tasks [75]. In another application, Aguirre [76] used the correlations between perceptual and distributed neural similarity matrices in color perception to illustrate the continuous carry-over design for fMRI experiments, which was developed for assessing the neural adaptation effects. In summary, the studies reviewed in this section illustrate the utility and flexibility of similarity analysis methods, particularly for investigating representation of objects linked to measures of neural activity.

### 4.2. Similarity of Individuals

Similarity analysis techniques can also be applied to an individuals × individuals proximity matrix. For example, MDS has been used as part of the algorithm for assessing group homogeneity [62, 77] and detecting outliers in fMRI data sets [62]. It has also been used to visualize proximities between participants based on mutual information [78] or the frequency of being classified in the same group as complementary information on the classification accuracy [39]. 

### 4.3. Similarity of Brain Regions

Another application of MDS focuses on the representational space of cortical areas. In one of the earlier applications of MDS to neuroimaging data, Friston et al. [79] examined the representational spaces of voxelwise connectivity during word generation tasks on healthy participants and patients with schizophrenia. Results suggested an abnormal prefrontal-temporal integration in schizophrenic groups. Resting state connectivity in healthy participants has been investigated with MDS on a connectivity matrix defined for anatomical regions of interest [31]. An INDSCAL variant was used to examine functionally connected brain regions in schizophrenia [6] and Asperger's Syndrome [59]. Shinkareva et al. [22] compared brain regions in terms of the confusion patterns based on the most likely prediction of the classifier for object classification. Hervé et al. [80] used MDS to visualize the similarity structure of interregional correlations in a study examining affective speech comprehension. Multidimensional scaling has also been used for visualization of coactivation relationships from meta-analyses [81]. In summary, similarity analysis techniques are useful in providing a visual representation of similarity of representations across neural regions.

## 5. Software

MDS and cluster analysis are implemented in most statistical packages. MATLAB (statistics toolbox), R, SAS, SPSS, and SYSTAT provide functions for classical and nonmetric multidimensional scaling and cluster analysis. Individual differences scaling is implemented in R with indscal() function in the SensoMineR package and the smacof package [82]. STATIS is implemented with statis() function in the ade4 package [83] in R. A highly versatile MATLAB toolbox for cluster analysis is available from Hubert et al. [84].

## 6. Summary

 We have reviewed the advantages and applications of different methods for examining similarity structures of activation patterns, along with potential cautions for interpreting these analyses. Using similarity as a level of analysis allows for comparison of representational structures across individuals, brain regions, and data collection methods. These analytic methods provide useful exploratory visualization tools. More importantly, used in conjunction with other methods of fMRI data analysis, similarity analysis methods provide a means for testing correspondence between similarity structure derived from imaging data and that derived from other sources, such as physical similarity or perceptual similarity. Similarity based methods, representational similarity analysis in particular, have been instrumental in examining hypotheses of neural representation of objects through comparison of internal representations derived from fMRI data to those derived from behavioral data and those derived from physical stimulus attributes. 
